# Molecular Docking Studies with Rabies Virus Glycoprotein to Design Viral Therapeutics

**DOI:** 10.4103/0250-474X.73905

**Published:** 2010

**Authors:** N. R. Tomar, V. Singh, S. S. Marla, R. Chandra, R. Kumar, A. Kumar

**Affiliations:** Department of Molecular Biology and Genetic Engineering, G. B. Pant University of Agriculture and Technology, Pantnagar-263 145, India; 1 Department of Veterinary Microbiology, G. B. Pant University of Agriculture and Technology, Pantnagar-263 145, India; 2 Animal Biotechnology Section, Central Sheep and Wool Research Institute, Avikanagar - 304 501, India

**Keywords:** Docking, glycoprotein, molecular operating environment, polyethylene glycol 4000, rabies, RVG

## Abstract

The genome of rabies virus encodes five proteins; the nucleoprotein, the phosphoprotein, the matrix protein, the glycoprotein, and the RNA-dependent RNA polymerase. Among these, the glycoprotein is the most important as it is the major contributor to pathogenicity and virus neutralizing antibody response. Keeping in mind that glycoprotein is the only protein exposed on the surface of virus and is thought to be responsible for the interaction with the cell membrane, it was attempted to target glycoprotein by a ligand polyethylene glycol 4000, which blocks its active site, as seen by molecular operating environment software, so that it may be possible to prevent the spread of virus into the host. The ligand polyethylene glycol 4000 was retrieved from Research Collaboratory for Structural Bioinformatics protein data bank by providing the glycoprotein sequence to the databank. In this study it was observed that the ligand was successfully docked on a major portion of antigenic site II of glycoprotein by mimicking the virus neutralizing antibodies. This knowledge may be important for the development of novel therapies for the treatment of rabies and other viral diseases in the future.

Rabies, severe encephalitis of mammals, is caused by the members of the lyssavirus genus of the *Rhabdoviridae* family, order *Mononegavirales*. The disease caused by rabies virus (RV) is fatal once clinical symptoms appear in the form of encephalomyelitis in several species of mammals including humans[1]. Human rabies is mainly transmitted through a rabid dog bite in the developing world, of which 94–98% of deaths are due to canine rabies[2]. World-wide human mortality from rabies is estimated to be 55 000 deaths per year with 56% of these deaths estimated to occur in Asia and 44% in Africa. In India alone, 18 500 people die and some 700 000 people take rabies prophylaxis each year following exposure to rabid animals.

The genome of virus is about 12 kb that encodes five proteins; the nucleoprotein (N), the phosphoprotein (P), the matrix protein (M), the glycoprotein (G), and the RNA-dependent RNA polymerase (L). The viral RNA, which is always encapsidated by N, forms the ribonucleoprotein (RNP), which is the template for viral replication and transcription[3]. The RVG, which is organized as a trimer, is the major contributor to pathogenicity. It interacts with cellular receptors[4], mediates pH-dependent fusion, and promotes viral entry from a peripheral site into the nervous system[5]. Moreover, RVG is involved in the trans-synaptic spread within the central nervous system[6,7]. Although RV pathogenicity is a multigenic trait, the G is major contributor to the pathogenicity of a particular RV[8]. The efficient interaction of RVG with putative host cell receptors can promote effective virus uptake resulting in increased virulence. There have been recent important advances in our understanding of how rabies virus spreads and causes disease in its hosts. Because current approaches to the management of human rabies have proven unsatisfactory, more research is needed in good experimental animal models in order for us to better understand the pathogenesis of this ancient disease.

Previous research addressing the control of sylvatic rabies has focused on the development of vaccines. Although a vaccine for rabies exists, the disease remains a problem. Some very good vaccines are available, but economic reasons have kept them from being used in developing countries. In these areas, animal vaccines for rabies are available but are not widely used, a situation that allows the virus to spread among domestic animals, enabling a reservoir of the virus to exist intimately with humans. In spite of great advances in virology there is yet no cure of this disease. The present study was attempted to dock the pathogenic glycoprotein with identification of a suitable ligand in order to search a cure for this disease by development of novel therapeutics based on the information of docked molecule with virus glycoprotein.

The modern docking programs/software packages e.g. MOE, Q-site finder, Ligand explorer, and Rasmol have been used to find the active site of the glycoprotein. The structure of ligand against this active binding site can be found by MOE. The exact conformation and configuration of the ligand can be calculated to find the best molecule with minimum binding energy and it can be used to develop potential drug molecules against the disease. If the glycoprotein can be targeted by a ligand which blocks its active site, it is possible to prevent the spread of virus into the host. This knowledge may be important for the development of novel therapies for the treatment of rabies and other viral diseases in the future.

## MATERIALS AND METHODS

In this investigation an attempt was made to carry out the docking of the ligand in to RVG protein with the following infrastructure. Intel^®^ Pentium 4, 1.8 GHz system having 256 MB RAM with the Microsoft Windows XP Pro 2002 operating platform was used. The software package MOE (Molecular Operating Environment)[9] was used for docking MOE is a software system designed by the Chemical Computing Group to support Cheminformatics, Molecular Modelling, Bioinformatics, Virtual Screening, Structure-based-design and can be used to build new applications based on SVL (Scientific Vector Language)

Rasmol[10] program was used to translate a PDB file containing detailed molecular structure information into a picture. RVG was used for docking with ligand polyethylene glycol 4000 (PE4). Q-site finder[11] tool was used to find out potential ligand binding sites on RVG molecule. RCSB Ligand explorer was used to visualize the interaction between RVG and Ligand.

### Preparation of ligand molecule:

**Fig. 1:**
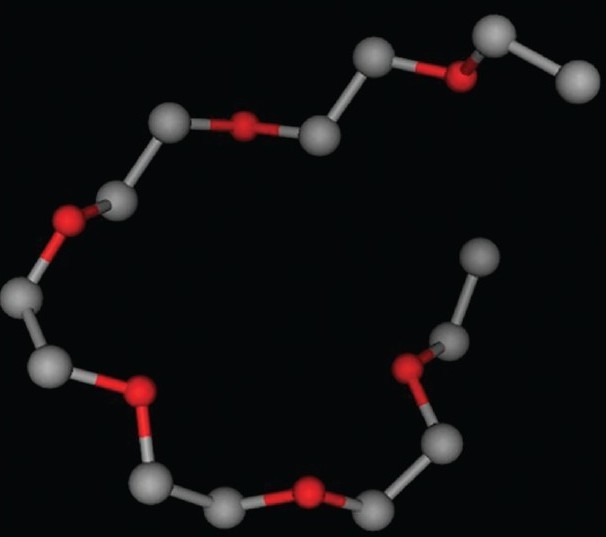
Ligand designing through CORINA

The structure of ligand polyethylene glycol 4000 (PE4) molecule was prepared using SMILE string from *Research Collaboratory for Structural Bioinformatics* (RCSB). Atomic coordinates were computed by the algorithm of the CORINA program (fig. 1).

### Preparation of receptor using homology modeling:

**Fig. 2:**
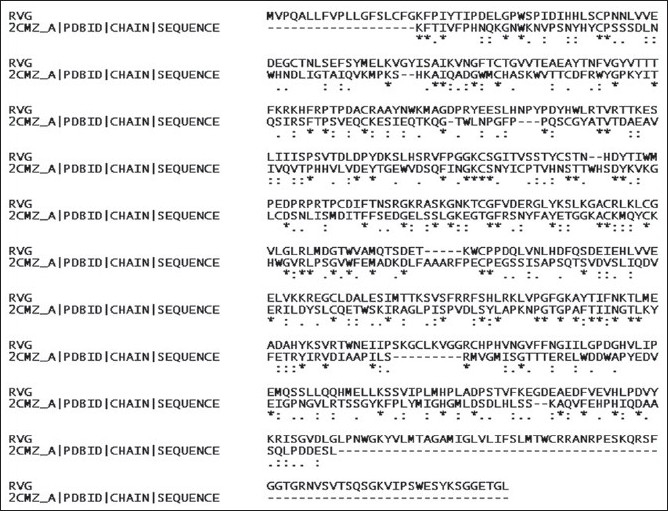
Alignment of RVG sequence with the template 2CMZ

For the preparation of receptor molecule, the protein sequence was loaded to the sequence editor tool and it was searched for template. The template 2CMZ was selected and used for the preparation of homology model. After loading the sequences these were aligned (fig. 2) by pairwise alignment using BLOSUM matrix and percent scoring method and the model was prepared by homology modeling using MMFF94X force field and RMS gradient of 0.5.

### Energy Minimization:

The energy of the protein molecule was minimized using the Energy minimization algorithm of MOE tool. The following parameters were used for energy minimization; gradient: 0.05, Force Field: MMFF94X+Solvation, Chiral Constraint: Current Geometry. Energy minimization was terminated when the root mean square gradient falls below the 0.05. The initial and final energy of protein were calculated (in kcal/mol) by GizMOE using MMFF94X force field with conjugant gradient method. The minimized structure was used as the template for Docking.

### Validation of modelled protein:

Validation of modelled structure was carried out using Structure Analysis and Verification Server[12]. Structure Analysis and Verification Server greatly simplifies computational analysis of the molecular structure and sequence of proteins. The stereochemical validation of model structures of proteins is an important part of the comparative molecular modeling process. The stereochemical quality of modeled protein was checked by Ramchandaran plot.

### Calculating the active site sequence:

Active sites present in the protein were identified from the 3D atomic coordinates of the receptor using Q-SITE FINDER. It is an energy-based method for the prediction of protein-ligand binding sites.

### Docking:

The binding of the ligand molecule with the protein molecule was analyzed using MOE docking program to find the correct conformation (with the rotation of bonds, structure of molecule is not rigid) and configuration (with the rotation of whole molecule, structure of the molecule remains rigid) of the ligand, so as to obtain minimum energy structure. The parameters used for the Docking were, Total Runs = 50, Cycle/Runs = 15, Iteration Limit=10 000, Potential Energy Grid: ON, Annealing Algorithm: Simulated Annealing.

## RESULTS AND DISCUSSION

The neuronal spread of rabies virus and hence the fusion of RV and host cell membrane should be controlled by blocking the active sites of the target glycoprotein. Conformational studies on the Asn194-Ser195-Arg196-Gly197 tetrapeptide, an essential part of the binding site of the rabies virus glycoprotein, indicate that the side chains of Asn and Arg could mimic the acetylcholine structure and is responsible for binding to the nicotinic acetyl choline receptor[13]. If a ligand could be designed in such a way that it could directly block such important sites, it may open the possibility to treat viral diseases.

**Fig. 3:**
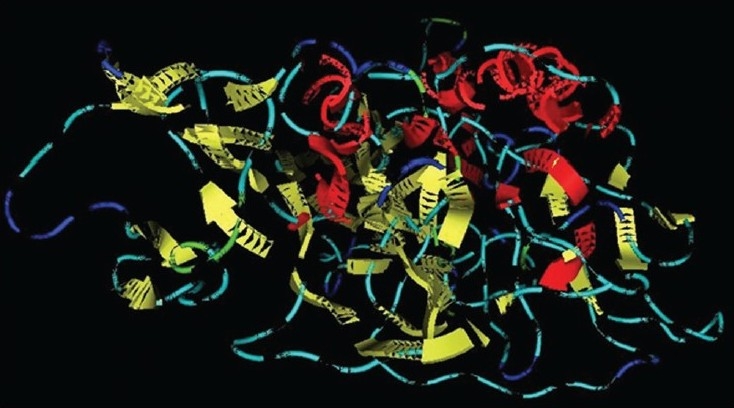
Preparation of receptor using homology modeling. The α-helix is represented by red, β-sheet by yellow and loops by sky blue.

**Fig. 4:**
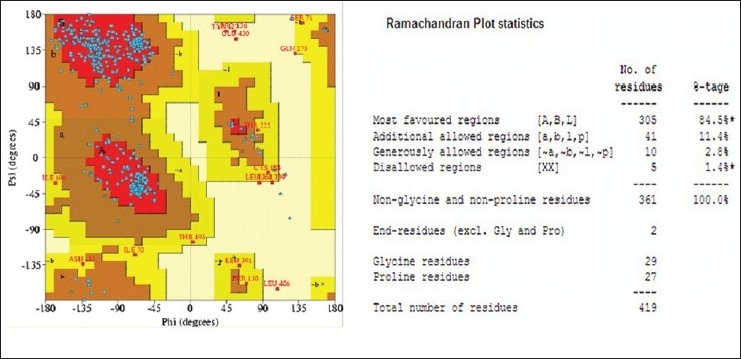
Ramachandran plot of modeled protein

By using our sequence of RVG from CVS strain (Accession number FJ979833) of rabies virus as a query sequence, many possible structures were generated by homology modeling using 2CMZ as a template. Among these the best structure (having minimum energy) was selected as a target of ligand PE4. The α-helix is represented by Red, β-sheet by yellow and loops by sky blue (fig. 3). Validation of modelled structures obtained from MOE was carried out using Structure Analysis and Verification Server. The structure modelled using MOE had over all quality factors of 60.741%. The stereochemical quality of the modelled protein was checked by ramchandran plot which showed 84.5% residues in most favoured regions and 11.4% residues in additionally allowed region (fig. 4).

The energy of the protein molecule was minimized using gradient value of 0.05 and MMFF94X force field. The initial energy of the protein molecule was 934.59 kcal/mol and the final energy was 661.1023 kcal/mol. The minimized structure was used as template for Docking. The active sites in the present study were found using Q-site finder tool. The purpose of Site Finder is to calculate possible active sites in a receptor from the 3D atomic coordinates of the receptor. Such a calculation is useful to determine potential sites for ligand binding in docking calculations. The 3D structures of the active site were visualized by using tools like Rasmol. The amino acid sequence was retrieved using MOE software and several ligand molecules were designed against the active site by targeting these amino acid.

**Fig. 5:**
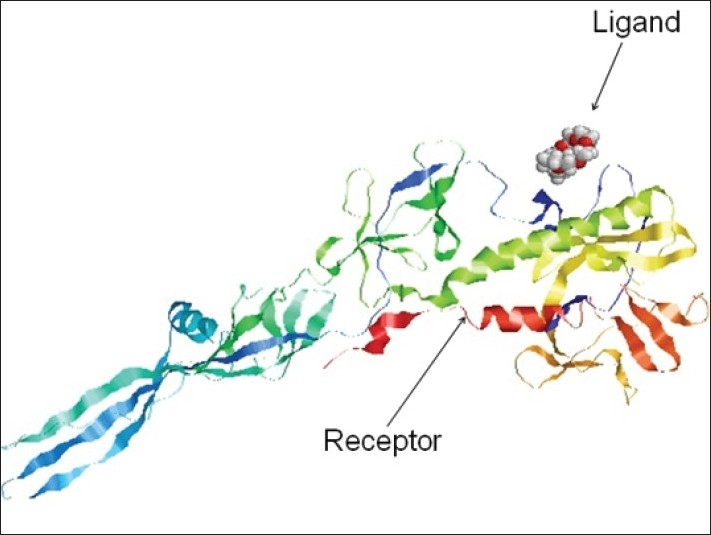
Docking of ligand PE4 with RVG Protein

The binding of the ligand molecule with the protein molecule was analyzed using MOE docking program (fig. 5). Docking studies gives different conformations of ligand from a single 3D conformation by applying a collection of preferred torsion angles to the rotatable bonds. For small ligands, a systematic search is conducted which generates all combinations of angles. For the docking procedure the pbd file of both ligand and glycoprotein were loaded into the MOE and then docking of the PE4 ligand with glycoprotein was done.

**Fig. 6:**
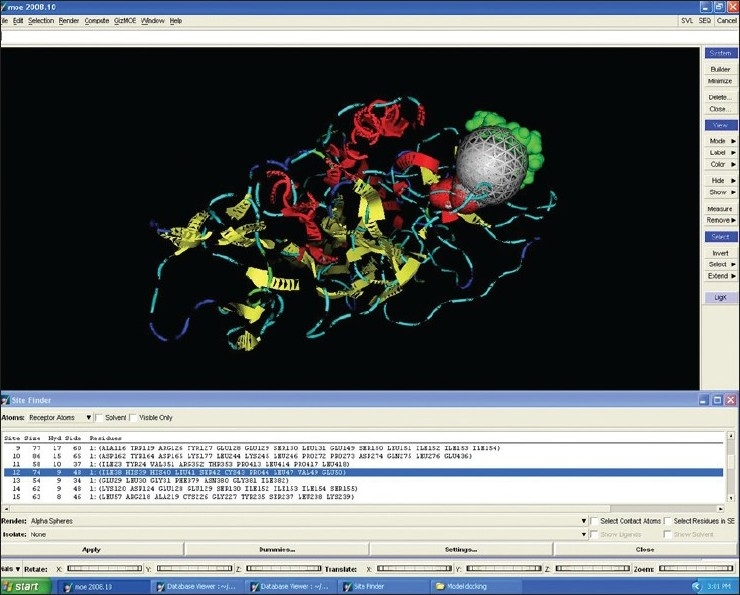
MOE window showing the active site of receptor

**Fig. 7:**
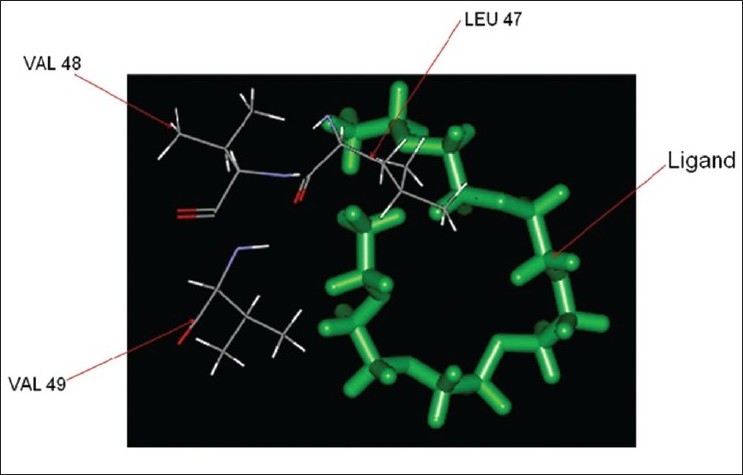
Ligand interaction with binding site atom and close residue

**Fig. 8:**
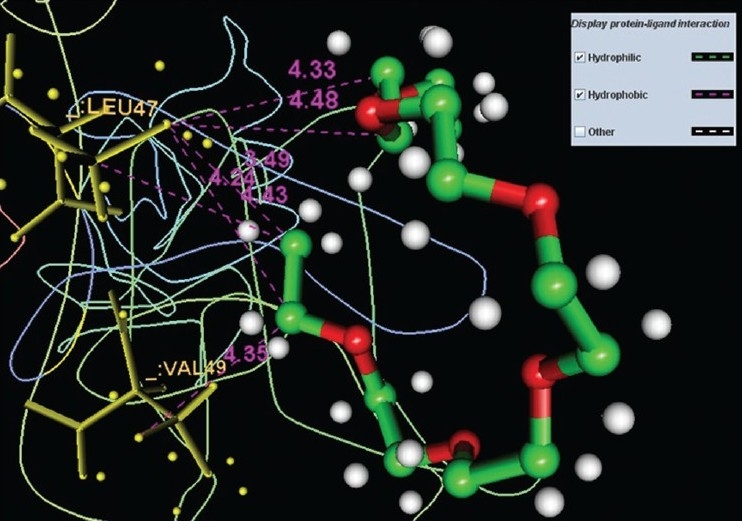
Hydrophobic interactions and distances between protein and ligand. Protein-ligand interactions are depicted by blue hatched lines for hydrophilic interactions and by pink hatched lines for hydrophobic interaction.

The pocket sequence of the active site was calculated by using site finder tool of MOE (fig. 6). From the site finder tool it is clear that the active site at which the ligand docks comprises Ile 38, His 39, His 40, Leu 41, Ser 42, cys 43, Pro 44, Leu 47, Val 49, Glu 50. The closest residues among them were 47-49 (fig. 7). The interactions between active site and ligand consist of hydrophobic and other interactions (fig. 8). The exact function of this region is not clear but some of its residues (Ile38 - Cys43) form the major portion of antigenic site II. This is responsible for the production of virus neutralizing antibodies. Instead of being docked on a site for viral pathogenesis, it docks on the antigenic site II. This is a unique site which not only provide access to the immune system for induction of protective antibody response but also a site for binding ligands which might act as potential suppressor molecules for inhibition of viral multiplication and pathogenesis of rabies virus. In this study it was observed that the ligand PE4 and the virus neutralizing antibodies docks on a common site. If this ligand could have a hampering effect on viral multiplication /pathogenesis by mimicking the virus neutralizing antibodies, it can be used to clear the virus from the circulation of patients which are having a defective immune response in which it is not able to produce virus neutralizing antibodies to clear the viruses from circulation. So this docking study promotes us to build new ligand(s) which can dock on the major antigenic sites for the clearance of pathogen from the body and also the ligands which can directly dock onto the regions responsible for the pathogenesis (from aa 194–197) of rabies virus to block the viral pathogenesis. The most important interactions which involve ligand and receptor’s active site are hydrogen bonding and ionic. These suggest that new ligand should be generated keeping in view that it should be able to have stronger hydrogen and ionic interaction with the amino acid moieties of the binding site. The smiles string of the ligands is achieved from MOE which can give the actual structure of the ligand. The chemical formula of PE4 can be found from RCSB which shall help us to build new ligand(s) to block the viral pathogenesis.

This ligand docks on a major portion of antigenic site II of glycoprotein. So this ligand may have a possibility to prevent the disease in patients suffering from immunodeficiency diseases. These docking studies promote us to build new ligand(s) which can completely block the viral pathogenesis by docking on the responsible sites.
